# C2H2 Zinc Finger Protein Family Analysis of *Rosa rugosa* Identified a Salt-Tolerance Regulator, *RrC2H2-8*

**DOI:** 10.3390/plants13243580

**Published:** 2024-12-22

**Authors:** Yong Xu, Yuqing Shi, Weijie Zhang, Kaikai Zhu, Liguo Feng, Jianwen Wang

**Affiliations:** 1 College of Horticulture and Landscape Architecture, Yangzhou University, Yangzhou 225009, China; yongxu@yzu.edu.cn (Y.X.); mz120221400@stu.yzu.edu.cn (Y.S.); b3771w@163.com (W.Z.); 2 Co-Innovation Center for Sustainable Forestry in Southern China, Nanjing Forestry University, Nanjing 210037, China; kkzhu@njfu.edu.cn

**Keywords:** *Rosa rugosa*, C2H2 zinc finger, salt tolerance, heterologous transformation

## Abstract

*Rosa rugosa* is a representative aromatic species. Wild roses are known for their strong tolerance to highly salty environments, whereas cultivated varieties of roses exhibit lower salt stress tolerance, limiting their development and industrial expansion. Previous studies have shown that C2H2-type zinc finger proteins play a crucial role in plants’ resistance to abiotic stresses. In this study, 102 C2H2-type zinc finger genes (*RrC2H2s*) were identified in *R. rugosa* via a comprehensive approach. These genes were categorized into three lineages, and their motif constitutions were grouped into four classes. *RrC2H2s* were distributed across all seven rose chromosomes, with 15 paralogous gene pairs identified within synteny regions. Additionally, 43 *RrC2H2s* showed differential expression across various tissues under salt stress, with *RrC2H2-8* being the only gene consistently repressed in all tissues. Subcellular localization analysis revealed that the RrC2H2-8 protein was localized in the nucleus. The heterologous expression of *RrC2H2-8* in *Arabidopsis* significantly improved its growth under salt stress compared to the wild-type (WT) plants. Furthermore, the malondialdehyde content in the roots of transgenic *Arabidopsis* was significantly lower than that in the WT, suggesting that *RrC2H2-8* enhanced salt tolerance by reducing cellular damage. This study provides a systematic understanding of the *RrC2H2* family and identifies *RrC2H2-8* as a regulator of salt tolerance, laying a foundation for future research on the mechanisms of salt stress regulation by *RrC2H2*.

## 1. Introduction

Salinity stress is one of the most serious abiotic stresses, adversely impacting the normal growth and development of plants and causing substantial economic losses in the ornamental flower industry [1,2]. *Rosa rugosa* is a deciduous shrub from the Rosaceae family, and it is among the oldest naturally aromatic plants worldwide. Its flowers are rich in aromatic compounds, such as terpenes, making it highly valuable for the spice industry [3,4]. To meet the growing market demand for rose flowers and their essential oil products, the cultivated area of *R. rugosa* in China has been expanding annually [4,5]. However, the limited salt tolerance of cultivated rose varieties restricts their widespread adoption and industrial production [6]. In contrast, wild *R. rugosa*, naturally distributed across the coastal regions of China, the Korean Peninsula, and Japan, is a halophyte with strong salt tolerance due to its adaptation to high-salinity environments [7,8]. This makes wild *R. rugosa* an important model for studying the mechanisms of salt tolerance in roses [9].

Zinc finger proteins (ZFPs) are characterized by their short, stable, finger-like structures that bind to Zn^2+^ ions through self-folding [10]. Based on the spatial arrangement of histidine and cysteine residues around the Zn^2+^ ion, ZFPs have been classified into several families, including C2H2, C2HC, C2HC5, C3HC4, C3H, C4, C4HC3, C6, and C8 [11]. The C2H2 family is among the largest plant transcription factor (TF) families, along with those relating to plant gene duplication events of ZFPs [10,12]. The C2H2 structure typically consists of 25–30 amino acids (CX_2-4_CX_3_FX_5_LX_2_HX_3-5_H) [13], with two cysteine residues and two histidine residues forming an α-helix and a double-stranded β-sheet, which serve as DNA-binding domains in the presence of Zn^2+^. C2H2 has been widely studied in regard to *Arabidopsis*, petunia, wheat, cotton, soybean, and rice, where they play crucial roles in leaf morphogenesis, flower development, and responses to both biotic and abiotic stresses [14,15,16,17,18,19]. Various approaches, including using Hidden Markov Models (HMM) from the Pfam database (http://pfam.xfam.org, accessed on 1 June 2023), self-constructed HMM, BLASTn searches (https://blast.ncbi.nlm.nih.gov/Blast.cgi, assessed on 1 June 2023), or manual selection, have been employed to identify plant C2H2 families [14,19,20]. However, the lack of standardized methodologies complicates cross-species identification, leading to inconsistencies. For instance, one study identified 176 AtC2H2 genes in *Arabidopsis* [19], of which genes have often been used as reference points in other research, whereas the PlantTFDB database (https://planttfdb.gao-lab.org/family.php?sp=Ath&fam=C2H2, accessed on 1 June 2023) predicts only 100 of these to be transcription factors. To address this, a comprehensive approach combining multiple methods would help reduce false positives and false negatives.

High salinity imposes serious stress on plant growth, and transcriptome analysis has identified hundreds of *C2H2* genes that are either upregulated or downregulated in response to salt stress in several plant species [21,22,23]. *C2H2* regulates plant salt tolerance by modulating the expression of stress-related genes involved in ion balance, antioxidant activity, and hormone signal transduction [12]. For example, salt stress induces the expression of *AtSIZ1* in *Arabidopsis*, and its overexpression increases K^+^, proline, and soluble sugar content while reducing Na^+^ and malondialdehyde (MDA) levels [24]. This suggests that *C2H2* genes may enhance salt tolerance by maintaining ion homeostasis and osmotic balance. Similarly, the *OsZFP179* gene in rice, which contains two C2H2 domains, significantly enhances salt tolerance when overexpressed [25]. This enhancement of salt tolerance is mediated through the induction of genes involved in ROS (reactive oxygen species) scavenging, ABA (abscisic acid)-responsive proline transporters, and the ABA-independent transcription factor *OsDREBA2A*, which is known for its role in salt tolerance. Additionally, *TaZNF* improves the salt tolerance of wheat by regulating Na^+^ excretion and stomatal apertures [26]. However, several *C2H2* genes have been proven to be negative regulators of salt tolerance, such as *MtZPT2-2* in *Medicago truncatula*, which negatively regulates salt tolerance by inhibiting high-affinity potassium transporter genes, thereby reducing Na^+^ loading into the xylem [27]. These findings highlight the dual role of C2H2 proteins in salt tolerance, which can involve both promoting ROS scavenging and enhancing osmotic potential [10,12].

In this study, we identified the *C2H2* family in *R. rugosa* through a comprehensive approach, combining BLASTn and HMM-based methods. We explored the classification, sequence characteristics, and gene duplication events of the *RrC2H2* family via phylogeny, synteny, and motif analyses. To investigate potential candidates involved in salt tolerance regulation, we constructed expression profiles of *RrC2H2* genes across various tissues under salt treatment. One key candidate, *RrC2H2-8*, was identified as a positive regulator of salt tolerance based on ectopic expression studies conducted on *Arabidopsis*. Our study provides a systematic understanding of the *RrC2H2* family and serves as a valuable foundation for future research on the mechanisms of salt stress regulation by *RrC2H2*.

## 2. Results

### 2.1. Lineages and Synteny of RrC2H2 Family

**Figure 1:**
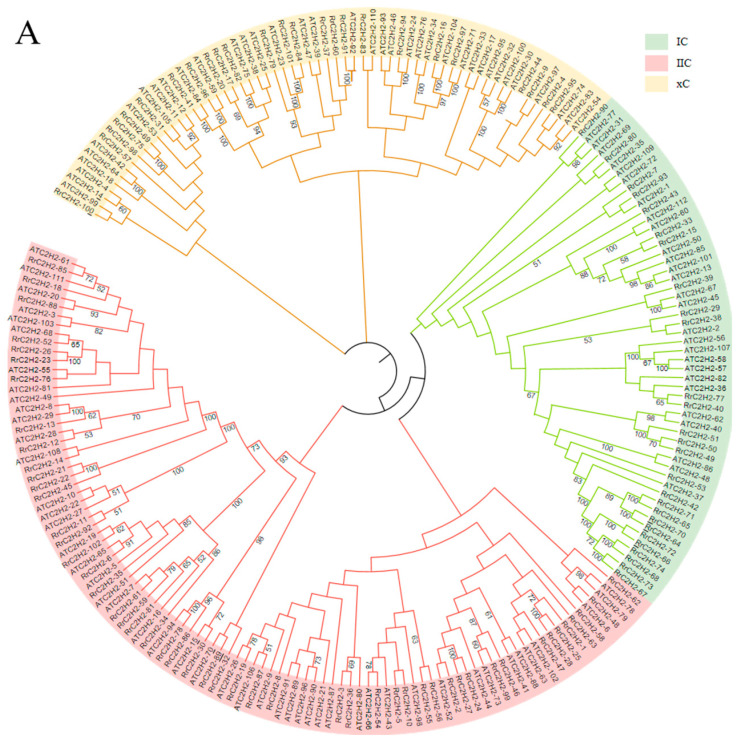

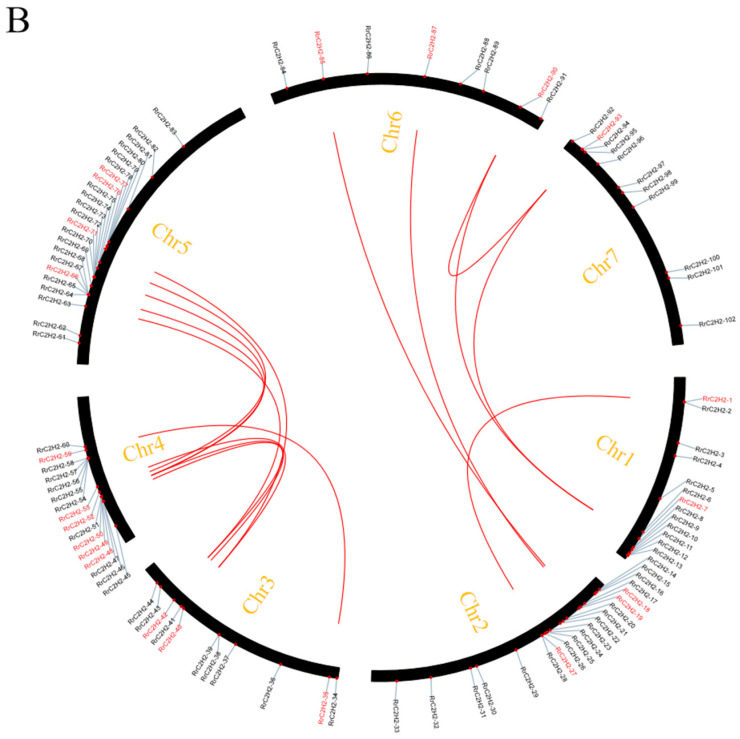
Phylogeny and intra-species synteny of *C2H2* family in *Rosa rugosa* (*RrC2H2*). (**A**) This dendrogram of the C2H2 family in *R. rugosa* and *Arabidopsis thaliana* was generated using the neighbor-joining method with 1000 bootstrap replicates. Branches were colored according to the three lineages. (**B**) The paralogous *RrC2H2s*, marked in red, are linked on the seven chromosomes by red lines.

The BLASTn search using reference *AtC2H2* genes identified more candidate *RrC2H2* genes than the HMM scan (Table S1). After verification via the PlantTFDB, CDD (NCBI Conserved Domains Database, https://www.ncbi.nlm.nih.gov, accessed on 28 June 2023), and PFAM databases, 102 *RrC2H2s* and 112 *AtC2H2s* were identified (Table S1). The members of the *RrC2H2* family were named sequentially based on their chromosomal locations in the rose genome (Figure S1). Notably, the number of *AtC2H2* genes was lower than the previously reported figure of 176 members. The neighbor-joining tree (NJ-tree) of the *C2H2* families in both *R. rugosa* and *Arabidropsis* classified 27, 20, and 25 *RrC2H2s* into lineage IC (one C2H2 domain), lineage IIC (two C2H2 domains), and lineage xC (three or more C2H2 domains), respectively (Figure 1A).

The *RrC2H2* genes are distributed across all seven rose chromosomes, with over 50 *RrC2H2* genes forming 11 distinct gene clusters (Figure S1). Tandem duplication events generated three gene clusters: *RrC2H2-48/RrC2H2-49/RrC2H2-50*, *RrC2H2-66/RrC2H2-67/RrC2H2-68* and *RrC2H2-72/RrC2H2-73/RrC2H2-74*. The whole-genome duplication or segmental duplication events resulted in 15 paralogous *RrC2H2* pairs in synteny regions on chromosomes Chr1-Chr6, Chr1-Chr7, Chr2-Chr6, Chr3-Chr4, Chr3-Chr5, Chr4-Chr5, and Chr6-Chr7 (Figure 1B).

### 2.2. Gene Structures and Conserved Motifs of the RrC2H2 Family

**Figure 2:**
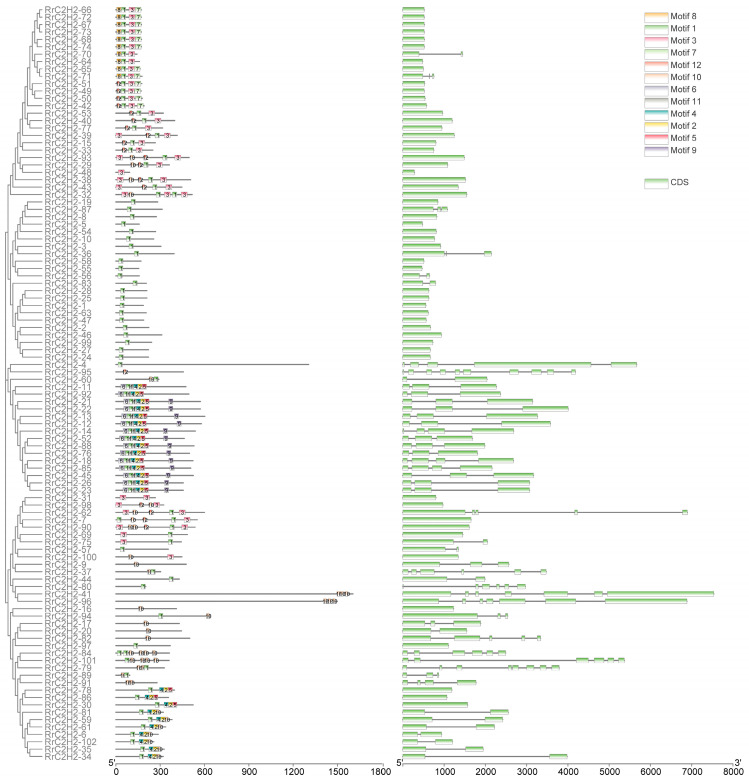
The conserved motifs and exon structures of the RrC2H2 family. The top 12 conserved motifs located using an amino acid scale plate are shown as boxes with numbers. The exons located using the nucleotide scale plate are represented by green boxes.

The length of RrC2H2 proteins varied from 93 to 1605 amino acids, with molecular weights ranging from 9.87 (RrC2H2-48) to 177.35 kDa (RrC2H2-41) (Table S1). The motif composition of RrC2H2 was categorized into four distinct groups: ‘motifs 8-1-3-7’, ‘motif 1’, ‘motifs 6-1-11-4-2-5’, and ’motif 10 containing’ (Figure 2). The last two groups corresponded to lineage xC, which contains three or more C2H2 domains, and these genes typically exhibited more complex structures with more introns (Figure 2).

### 2.3. Expression Analysis Regarding RrC2H2s

**Figure 3:**
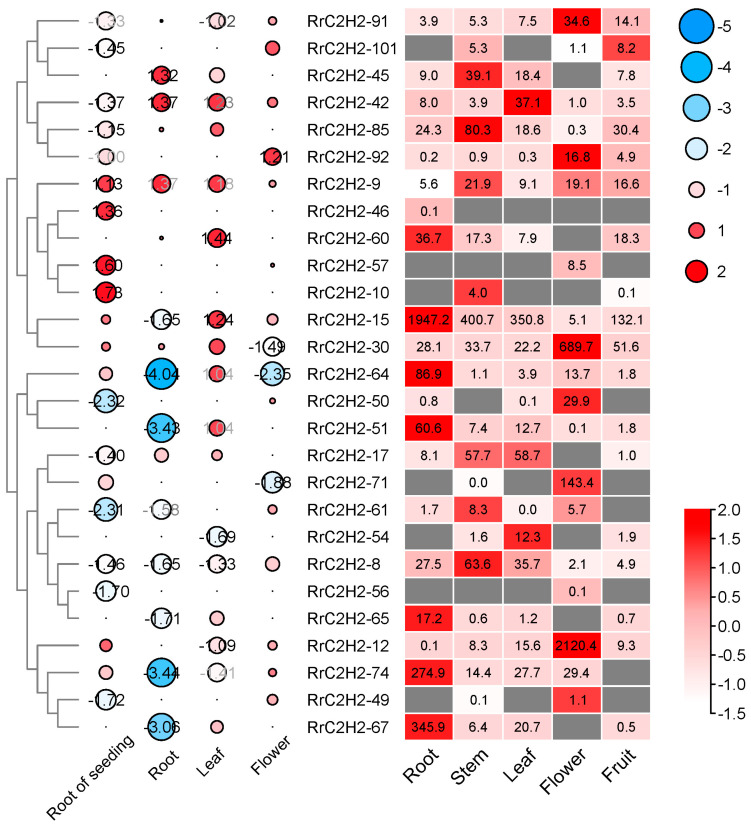
Expression profiles of differentially expressed *RrC2H2s* under salt stress in different tissues of *R. rugosa*. Circles indicate Log_2_ (fold changes) in the expression of *RrC2H2s* in the young roots of seedlings and the flowers, leaves, and roots of mature plants treated with 340 mM of NaCl for 1 h compared to water-treated controls. The boxes show normalized FPKM (the fragments per kilobase of exon model per million mapped frag-ments) values, with dark boxes indicating no RNA-seq reads were detected. Rows are clustered based on the hierarchical clustering of Log_2_ (foldchanges).

An expression profile analysis of five tissues of *R. rugosa* excluded 25 low-abundance *RrC2H2s* that were not expressed in any tissues (Figure S2, Table S2). An analysis of differentially expressed genes identified 4, 11, 11, and 17 *RrC2H2s* that responded to salt stress in the flowers, leaves, and roots of mature rose plants and young roots of rose seedlings, respectively (Figure 3, circles, Table S3). Among these, 1, 6, 3, and 6 *RrC2H2s* were induced by salt, while 3, 5, 8, and 11 *RrC2H2s* were repressed by salt in flowers, leaves, roots, and young roots, respectively. Notably, *RrC2H2-8* and *RrC2H2-9* exhibited consistent induction or repression across all tissues, whereas other genes showed divergent patterns in different tissues. The expression of *RrC2H2-8* (evm.model.Chr1.4174) in rose roots under salt stress was verified using quantitative real-time polymerase chain reaction (qRT-PCR) (Figure S3A). Additionally, under ABA treatment, only *RrC2H2-8* displayed significant repression within 1 h, a finding consistent with the downregulation pattern observed under salt stress (Figure S3B).

### 2.4. Subcellular Localization of RrC2H2-8

**Figure 4:**
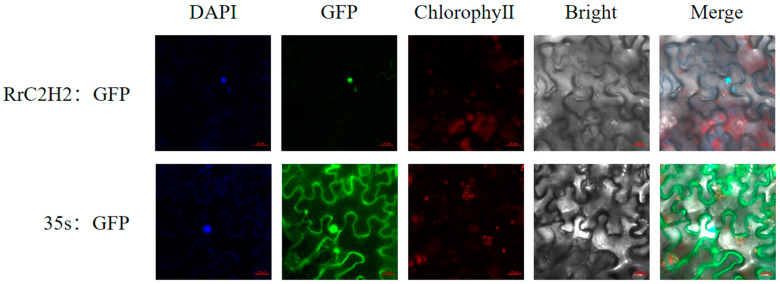
The subcellular localization of RrC2H2-8. Green fluorescent protein (GFP) empty vector (pGreen-SubC, 35s: GFP) and pGreen-SubC-RrC2H2-8 vectors were transiently transformed into *Nicotiana benthamiana* leaf epidermis and observed using laser confocal microscopy. Merged images (Merge) combine bright fields (Bright), fluorescence of chloroplast channel (ChlorophyII), nucleus marker channel (DAPI), and GFP channel in dark fields.

All RrC2H2 proteins were predicted to localize in the nucleus, except for RrC2H2-95, which was predicted to be localized in both the mitochondria and nucleus. RrC2H2-8 contained a single C2H2 zinc finger, and its C-terminal *α*-helix overlapped with the NLS (nuclear localization signal) sequence ‘SSQALGGHQNAHKRERTMAKRALRM’ (Figure S4). The co-localization of green fluorescence from the RrC2H2-8-GFP fusion protein and blue fluorescence from DAPI (4′,6-diamidino-2-phenylindole) in *Nicotiana benthamiana* leaves confirmed this protein’s nuclear localization (Figure 4).

### 2.5. Ectopic Expression of RrC2H2-8-Enhanced Salt Tolerance

**Figure 5:**
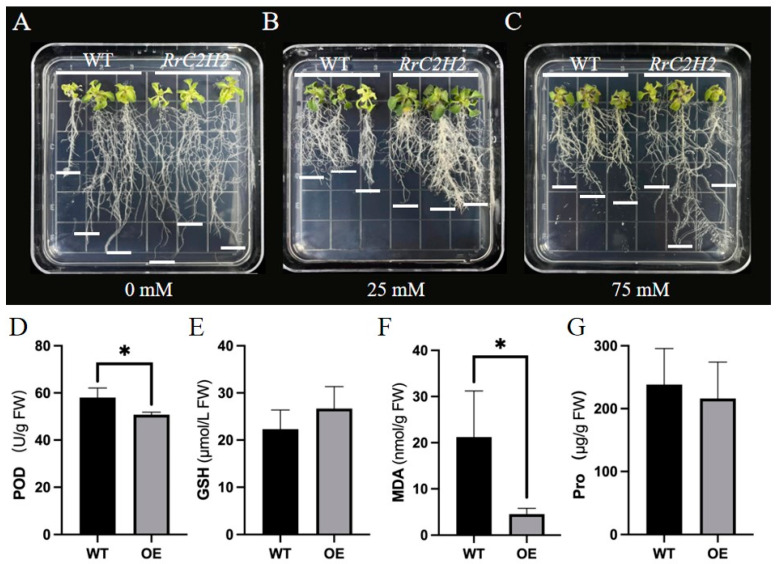
Salt-tolerance phenotypes and biochemical assays of WT and *RrC2H2-8* transgenic A. thaliana lines (OE). (**A**–**C**) Phenotypic observations under 0, 25, and 75 mM NaCl treatments. (**D**–**G**) Peroxidase (POD), reduced glutathione (GSH), malondialdehyde (MDA), and proline (Pro) contents in roots. Note: Asterisks denote statistically significant differences (* *p* < 0.05).

Positive homozygous lines (T3 generation) of *Arabidopsis thaliana* were verified for the insertion of *RrC2H2-8*, and the top three lines with high expression levels were selected for salt treatment. Under non-saline conditions (1/2 MS medium), the growth of the wild-type (WT) and transgenic lines (OE) was similar, with no significant differences (Figure 5A). However, under 25 mM NaCl, the growth of the WT lines was significantly inhibited compared to that of the transgenic lines, which displayed robust roots (Figure 5B). At higher salt concentrations (75 mM NaCl), the WT seedlings showed wilting and yellowing of their leaf tips, indicating severe stress, while the transgenic lines displayed notably stronger tolerance, as evidenced by their longer roots and healthy leaves (Figure 5C). These results suggested that ectopic expression of *RrC2H2-8* increased the salt tolerance of *A. thaliana*. Additionally, the contents of peroxidase (POD), reduced glutathione (GSH), MDA, and proline (Pro) in the roots of WT and OE under the 75 mM NaCl treatment were analyzed (Figure 5D–G). Notably, MDA levels decreased significantly (with a foldchange of 4.7) in the OE compared to those of the WT, indicating that *RrC2H2-8* may regulate salt tolerance by protecting root cells from NaCl-induced damage.

## 3. Discussion

In this study, we examined the *R. rugosa RrC2H2* family, consisting of 102 members, a number comparable to the *AtC2H2* family [19], indicating that the *C2H2* zinc finger gene family is relatively evolutionarily conserved across species [20,21]. This family was categorized into three distinct lineages based on the number of zinc finger domains, with the conserved motif group corresponding to the triple helix domain showing the highest degree of conservation. Among these, *RrC2H2-8*, localized in the nucleus, exhibited significant repression under salt stress. This repression appears to play a critical role in mitigating cellular damage, supporting the hypothesis that *RrC2H2-8* acts as a positive regulator of salt stress tolerance in wild roses.

Although ZFPs are commonly recognized as TFs that bind DNA, some ZFPs can also mediate stress responses by interacting with RNA or other proteins. For example, the interaction of ZFP SERRATE with the double-stranded RNA protein HYL1 is involved in miRNA biogenesis, including the regulation of stress-responsive miRNAs [28]. Similarly, interactions between ZAT7 and HASTY enhance plant salt tolerance [29], and OsbZIP23 interacts with OsGF14 to positively modulate osmotic stress tolerance in rice [30]. These findings illustrate that ZFPs play multifaceted roles beyond transcription regulation. The highly conserved ‘QALGGH’ motif, specific to plant zinc finger domains [12,31], was identified within the NLS of RrC2H2-8 and other RrC2H2 members, suggesting that this motif contributes to the subcellular localization of plant ZFPs. STZ, the first AtZFP linked to salt tolerance, complements the sensitivity of calcineurin-deficient yeast and is induced by salt [32]. Since ABA plays a pivotal role in plant responses to osmotic and salt stress [32], many ABA-responsive ZFPs exhibit induction kinetics similar to salt-responsive genes [33]. For instance, *RrC2H2-8* is homologous to *ZFP4 (AtC2H2-21)* and *ZFP7 (AtC2H2-9)*, both of which act as negative regulators of ABA signaling and photomorphogenesis in *A*. *thaliana*, alongside *ZFP1* and *ZFP3* during seed germination [34,35]. Interestingly, the repression of *RrC2H2-8* in rose roots following ABA treatment parallels the inhibition of *ZFP3* during germination under ABA. Notably, the rapid repression of *RrC2H2-8* observed within 1 h suggests that its response to ABA may be part of an early upstream signal in the salt-stress response pathway.

The minimal phenotypic differences observed between the wild-type and *zfp3* mutant or silenced *ZFP1, ZFP4*, and *ZFP7* lines suggest a potential functional redundancy among ABA-related ZFPs [34,35]. *RrC2H2-8* clusters with *RrC2H2-19* and *RrC2H2-87* within the same phylogenetic branch, indicating possible gene redundancy. However, unlike prior reports of plant growth inhibition due to *ZFP3* overexpression [34,35], lines overexpressing *RrC2H2-8* exhibit germination and growth phenotypes similar to those of the WT. This discrepancy suggests that the regulatory roles of homologous ZFP3 differ from those of *RrC2H2-8* in roses. Additionally, the observation of stronger roots with lower MDA levels indicates that the ectopic expression of RrC2H2-8 protects root cells from salt stress damage in *Arabidopsis*. In *Arabidopsis*, *ZAT10* enhances the activity of ROS -scavenging enzymes, such as ascorbate peroxidase1 (APX1), APX2, and Fe-superoxide dismutase1 [36]. Similarly, *SlZF3* contributes to ascorbic acid biosynthesis in transgenic tomatoes [37]. However, in our study, no significant differences in POD and GSH content were observed between *RrC2H2-8*-overexpressing lines and the WT, suggesting that *RrC2H2-8* should not regulate the ROS scavenging system in *R. rugosa*. Additionally, *OsZFP252* and *OsZFP179* in rice [25,38], *IbZFP1* in sweet potato [39], and *AtZFP3* [34] and *AtSIZ1* in *Arabidopsis* [24] enhance salt tolerance by increasing the content of osmoregulatory agents, including Pro, soluble sugars, and stress proteins. Nevertheless, the absence of significant Pro accumulation in the *RrC2H2-8*-overexpressing lines implies that *RrC2H2-8* may not regulate osmolyte content in roses.

It has been reported that certain *ZFPs*, such as *MtZPT2-2* in alfalfa [27], *ZmC2H2-149* in maize [40], and *MdZAT10* in apple [41], act as negative regulators of stress tolerance, being suppressed under salt stress or ABA. Therefore, the apparent contradiction between the downregulation of *RrC2H2-8* under salt stress and its role in promoting salt tolerance requires further study. Given the availability of CRISPR-Cas9 tools for studying gene regulation in roses [42], applying this technology to knock out *RrC2H2-8* in the rose hairy root system could provide valuable insights into its regulatory role in salt stress responses and ABA sensitivity.

## 4. Materials and Methods

### 4.1. Identification and Phylogenetic Analyses of RrC2H2s Family

The *R. rugosa* genome was obtained from GDR (Genome Database for Rosaceae, https://www.rosaceae.org/, accessed on 20 June 2023). *C2H2* reference genes of *A. thaliana* were obtained from PlantTFDB. Candidate proteins were screened via BLASTn hits based on At*C2H2* sequences and through HMMER search based on the C2H2 domain (PF13912, http://Pfam.sanger.ac.uk/, accessed on 20 June 2023) in the *R. rugosa* genome, with a cutoff threshold value of <10^−5^. Ambiguous or incomplete candidates were excluded after verification using the Pfam PlantTFDB, and CDD database.

Based on the alignment of the C2H2 full-length protein sequence using Multiple Alignment using Fast Fourier Transform (MAFFT) [43], a NJ-tree was constructed using MEGA-X (https://www.megasoftware.net/, accessed on 5 July 2023), with 1000 bootstrapping replications [44]. The p-distance substitution model was used for phylogenetic analysis, with pairwise deletion applied to gaps and missing data, and uniform evolutionary rates were assumed across all sites.

### 4.2. Synteny Analysis and Motif Analysis of RrC2H2s Family

Homologous gene pairs within the *R. rugosa* genome (*E* < 10^−5^, top five matches) were identified through BLASTP search (https://blast.ncbi.nlm.nih.gov/Blast.cgi?CMD=Web&PAGE_TYPE=BlastHome, assessed on 10 June 2023). MCScanX was used to identify syntenic regions and predict gene duplication events based on the locations of homologous gene pairs [45]. *RrC2H2s* and their homologous counterparts in syntenic regions were visualized using Basic Circos of TBtools [46]. The top 12 conserved motifs were predicted using MEME web tools (https://meme-suite.org/meme/, accessed on 10 July 2023) with default parameters. Gene structures and motifs were visualized using the Gene structure view tool available with TBtools [46].

### 4.3. Expression Analysis Under Salt Stress and ABA Treatment

FPKM (the fragments per kilobase of exon model per million mapped fragments) values for *RrC2H2s* and the fold-changes of differentially expressed *RrC2H2s* were derived from our previous transcriptome and common *R. rugosa* database through expression analysis [9,47,48,49].

To verify the expression of *RrC2H2-8* in rose roots under salt stress, qRT-PCR assays were performed. One-month-old wild *R. rugosa* seedlings were treated with 340 mM NaCl solution for 1 h, and root samples were collected, with three biological repetitions. Total RNAs were extracted using the RNAprep Pure plant kit (Tiangen, Beijing, China) and reverse-transcribed into cDNA templates using HiScript^®^ III RT SuperMix (Vazyme, Nanjing, China). qRT-PCR was conducted using ChamQ SYBR Color qPCR Master Mix (Vazyme, Nanjing, China) on the CFX96 platform (Bio-Rad, Hercules, CA, USA). All procedures, including RNA extraction, reverse transcription, and qRT-PCR, were conducted following the manufacturer’s protocols. Primer sequences for *RrC2H2-8* and the reference gene are listed in Table S4. Furthermore, to investigate the response of *RrC2H2-8* to ABA, one-month-old plants (18 total) were treated with 15 mL of 0.1 mM ABA solution via spraying. Root samples were collected at 0, 1, 3, 6, 12, and 24 h post-treatment, with three biological repetitions for each time point. The expression levels of *RrC2H2-8* were analyzed using qRT-PCR, as described above.

### 4.4. Subcellular Localization Analysis

Subcellular localization of proteins was predicted using the web tools WoLF PSORT (https://wolfpsort.hgc.jp/, accessed on 20 October 2023) and Plant-mPLoc (http://www.csbio.sjtu.edu.cn/, accessed on 20 October 2023) based on their amino acid sequences [50,51]. The NLS of *RrC2H2-8* was predicted using the NLSExplorer web tool (http://www.csbio.sjtu.edu.cn/bioinf/NLSExplorer/, accessed on 20 October 2023) [52]. The coding sequence of *RrC2H2-8* was cloned into the subcellular localization vector pGreen-SubC. RrC2H2-8-GFP fusion proteins were transiently expressed in tobacco (*N. benthamiana*) leaves via Agrobacterium transformation. Fluorescence from GFP, DAPI, and chloroplasts in tobacco epidermal cells was observed using laser confocal microscopy.

### 4.5. RrC2H2-8 Overexpression and Salt-Tolerance Observation

The coding sequence of *RrC2H2-8* was subcloned into the stable overexpression vector (pNC-CAM1304-MCS35s) and transformed into *Agrobacterium tumefaciens* strain EHA105 using the liquid nitrogen freeze–thaw method. The EHA105-mediated genetic transformation of *A. thaliana* and the selection of T3-generation transgenic plants using hygromycin resistance were conducted as described previously [53]. The genomic insertion of *RrC2H2-8* in transgenic plants was verified with the Plant Direct PCR Kit (Vazyme, Nanjing, China), and its overexpression levels were confirmed using qRT-PCR. Overexpression lines were grown on 1/2 MS medium for 7d before transplantation onto 1/2 MS medium supplemented with 0, 25, and 75 mM NaCl for 14d, respectively.

After phenotype observation, the contents of POD, GSH, Pro, and MDA in the roots of transgenic plants were quantified using kits (Jiancheng, Nanjing, China) according to the manufacturer’s protocols. Absorbance values were measured at 420 nm, 450 nm, 520 nm, and 530 nm using a Bio-Tek Synergy 2 microplate reader (Agilent, Wilmington, DE, USA), respectively.

## 5. Conclusions

In this study, a comprehensive analysis using BLASTn and HMM-based methods identified 102 *RrC2H2s* in *R. rugosa*. These genes exhibited substantial variation in molecular weight and were grouped into three classes based on the number of C2H2 domains. The motif composition was categorized into four classes. *RrC2H2s* were distributed across all seven rose chromosomes, with 15 paralogous gene pairs within synteny regions. Among 43 differentially expressed *RrC2H2s* under salt stress, *RrC2H2-8* was consistently repressed across all tissues and localized in the nucleus. Transgenic *Arabidopsis* overexpressing *RrC2H2-8* exhibited improved growth and reduced MDA levels under salt stress, suggesting that *RrC2H2-8* enhances salt tolerance by reducing cellular damage. In conclusion, in this study, we systematically characterized the *RrC2H2* family and confirmed *RrC2H2-8* is a positive regulator of salt tolerance.

## Data Availability

The original contributions presented in the study are included in the article/supplementary material, further inquiries can be directed to the corresponding author.

## Supplementary Materials

The following information is available online at https://www.mdpi.com/article/10.3390/plants13243580/s1. Figure S1: Chromosomal locations of the *RrC2H2* gene family in rose; Figure S2: Heatmap of *RrC2H2* gene expression in different tissues of rose; Figure S3: Relative expression of *RrC2H2-8* in rose roots under salt and ABA treatments; Figure S4: The NLS motif and C2H2 domain of the RrC2H2-8 protein; Table S1: Identification of *C2H2* gene family in *R. rugosa* and *Arabidopsis*; Table S2: FPKM values of *RrC2H2* family members across different tissues in *R. rugosa*; Table S3: Differentially expressed *RrC2H2s* in *R. rugosa*; Table S4: Primers for qRT-PCR analysis.
