# Supplementary material for: C2H2 Zinc Finger Protein Family Analysis of Rosa rugosa Identified a Salt-Tolerance Regulator, RrC2H2-8
Source: Plants (Basel). 2024 Dec 22;13(24):3580. doi: 10.3390/plants13243580 (PMC11678247; doi:10.3390/plants13243580)
Supplement: Supplementary file 1 [file plants-13-03580-s001.zip › Figure S1-S4.pdf]

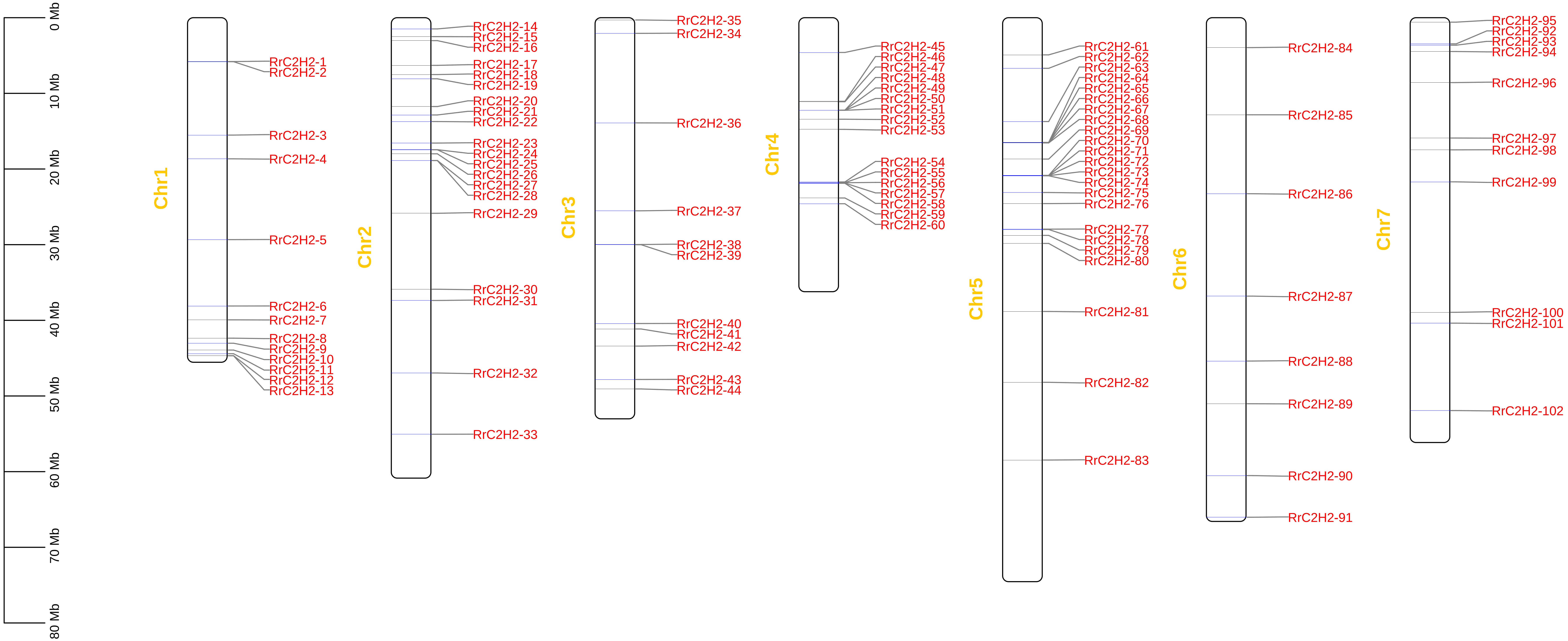

Figure S1. Chromosomal locations of the *RrC2H2* gene family in rose.

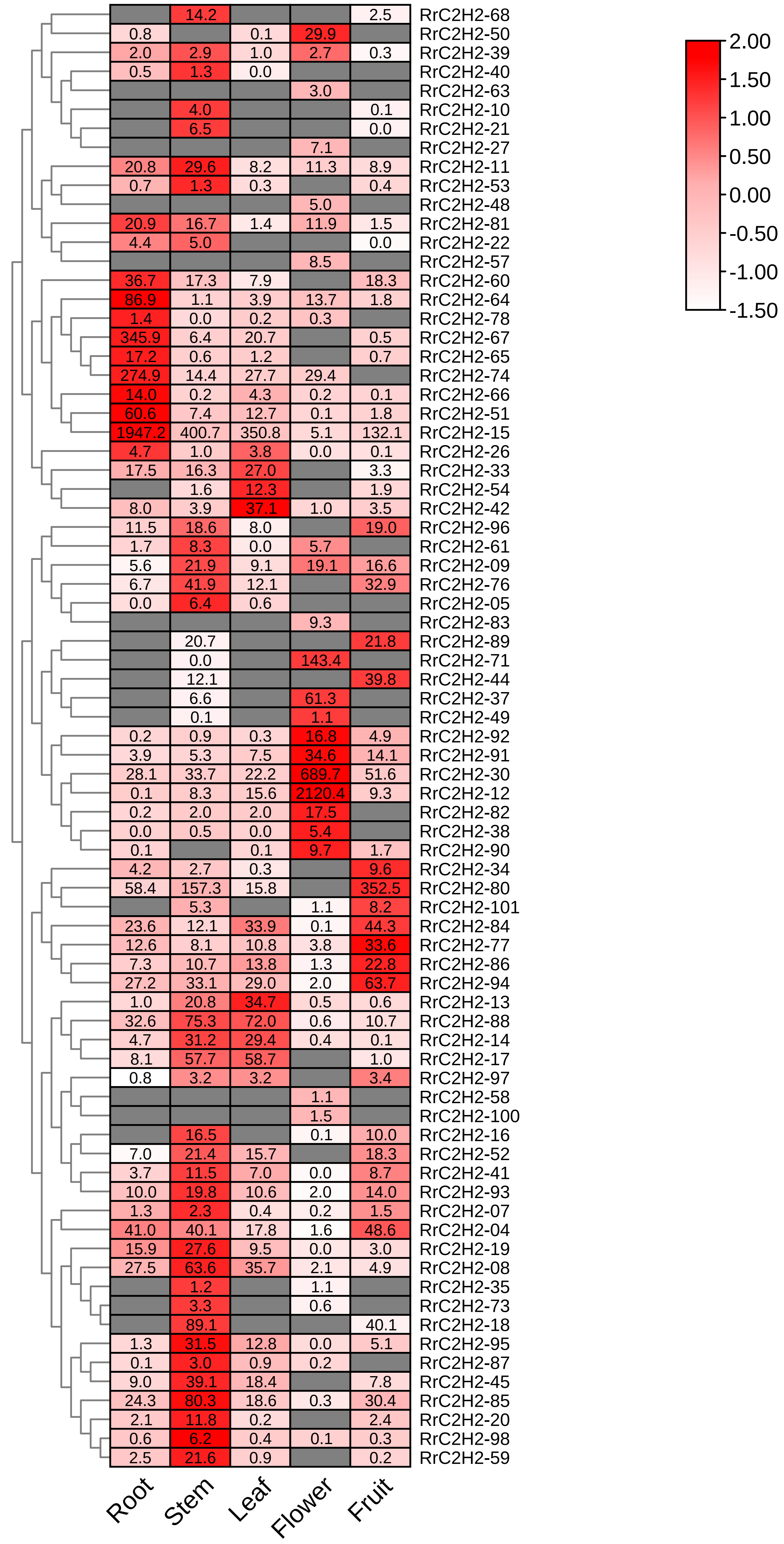

Figure S2. Heatmap of *RrC2H2* gene expression in different tissues of rose. The columns represent various tissues (root, stem, leaf, flower, and fruit), while the rows correspond to individual genes. Box colors indicate normalized expression levels, and the numbers represent FPKM values.

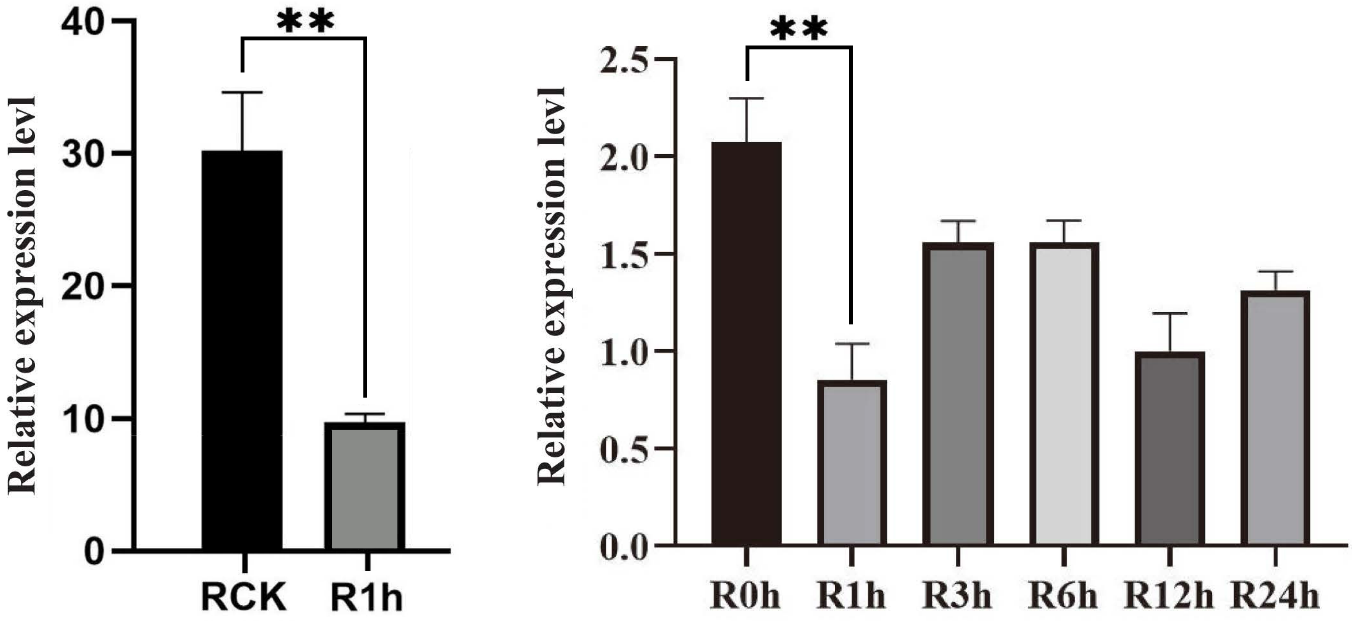

Figure S3. Relative expression of *RrC2H2-8* in rose roots under salt (a) and ABA treatments (b). RCK represents the roots of the control group without salt treatment; R0h, R1h, R3h, R6h, R12h, and R24h indicate the roots of groups subjected to ABA or salt treatments at 0, 1, 3, 6, 12, and 24 hours, respectively. Asterisks denote statistically significant differences (** $P < 0.01$).

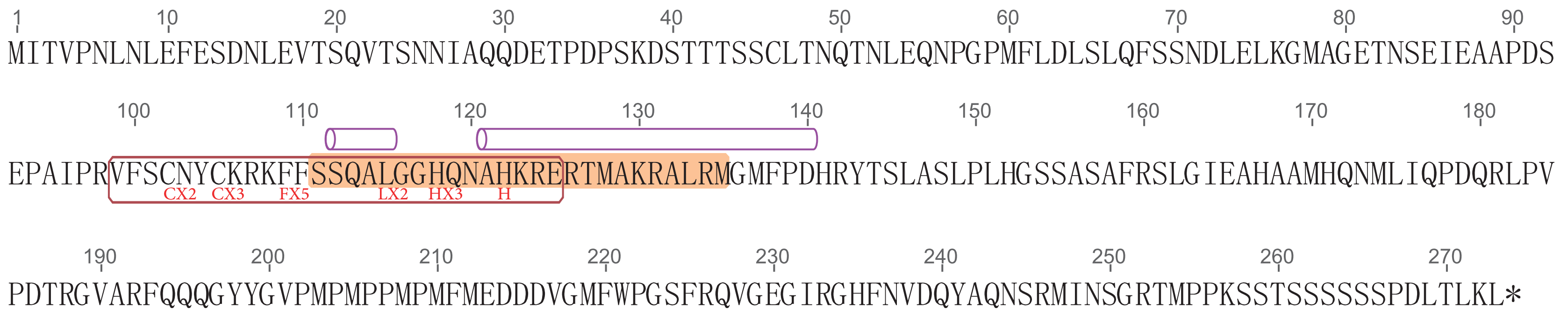

Figure S4. The NLS motif (highlighted) and C2H2 domain (boxed) of the RrC2H2-8 protein. Helical structures are indicated by cylinders above the sequence.
